# Correction: Słoniewski et al. A Comparative Analysis of Quality of Life in Women Diagnosed with Breast and Ovarian Cancer. *Int. J. Environ. Res. Public Health* 2022, *19*, 6705

**DOI:** 10.3390/ijerph22060961

**Published:** 2025-06-19

**Authors:** Robert Słoniewski, Marta Dąbrowska-Bender, Urszula Religioni, Adam Fronczak, Anna Staniszewska, Aneta Duda-Zalewska, Magdalena Milewska, Magdalena Kędzierska, Rafał Adam Matkowski, Grażyna Dykowska, Anna Słoniewska, Anna Kupiecka

**Affiliations:** 1 Department of Public Health, Medical University of Warsaw, 02-091 Warsaw, Poland; adamum@op.pl (A.F.); aneta.duda-zalewska@wum.edu.pl (A.D.-Z.); 2 Department of Clinical Dietetics, Medical University of Warsaw, 02-091 Warsaw, Poland; magdalena.milewska@wum.edu.pl; 3 School of Public Health, Centre of Postgraduate Medical Education of Warsaw, 01-813 Warsaw, Poland; urszula.religioni@gmail.com; 4 Department of Experimental and Clinical Pharmacology, Medical University of Warsaw, 02-091 Warsaw, Poland; anna.staniszewska@wum.edu.pl; 5 Department of Chemotherapy, Medical University of Lodz and Copernicus Memorial Hospital, CCC & T, 90-419 Lodz, Poland; kameleonmagda6@gmail.com; 6 Department of Oncology, Wrocław Medical University, Lower Silesian Oncology, Pulmonology and Hematology Center, 53-413 Wroclaw, Poland; rafal.matkowski@dcopih.pl; 7 Department of Health Economics and Medical Law, Medical University of Warsaw, 02-091 Warsaw, Poland; grazyna.dykowska@wum.edu.pl; 8 Masovian Oncological Hospital, 05-135 Wieliszew, Poland; sloniewska.anna@gmail.com; 9 OnkoCafe Foundation—“Together Better”, 04-175 Warsaw, Poland; biuro@onkocafe.pl

Marta Dąbrowska-Bender was not listed as co-corresponding author and co-first author in the original publication [1]. Robert Słoniewski and Marta Dąbrowska-Bender contributed equally to the publication and are both first authors. The authorship has been corrected accordingly. The authors state that the scientific conclusions are unaffected. This correction was approved by the Academic Editor. The original publication has also been updated.
